# The Effects of Different Stages of Mindfulness Meditation Training on Emotion Regulation

**DOI:** 10.3389/fnhum.2019.00208

**Published:** 2019-06-27

**Authors:** Qin Zhang, Zheng Wang, Xinqiang Wang, Lei Liu, Jing Zhang, Renlai Zhou

**Affiliations:** ^1^Department of Psychology, Nanjing University, Nanjing, China; ^2^College of Teacher Education, Qilu Normal University, Jinan, China; ^3^School of Psychology, Beijing Normal University, Beijing, China; ^4^Department of Psychology, College of Teacher Education, Ningbo University, Ningbo, China

**Keywords:** mindfulness meditation, focusing attention, open monitoring, emotion regulation, Stroop task

## Abstract

This study examined mood enhancement effects from 4-week focusing attention (FA) meditation and 4-week open monitoring (OM) meditation in an 8-week mindfulness training program designed for ordinary individuals. Forty participants were randomly assigned to a training group or a control group. All participants were asked to perform cognitive tasks and subjective scale tests at three time points (pre-, mid-, and post-tests). Compared with the participants in the control group, the participants in the meditation training group showed significantly decreased anxiety, depression, and rumination scores; significantly increased mindfulness scores; and significantly reduced reaction times (RTs) in the incongruent condition for the Stroop task. The present study demonstrated that 8-week mindfulness meditation training could effectively enhance the level of mindfulness and improve emotional states. Moreover, FA meditation could partially improve individual levels of mindfulness and effectively improve mood, while OM meditation could further improve individual levels of mindfulness and maintain a positive mood.

## Introduction

Mindfulness can be defined as nonjudgmental attention to the present moment (Kabat-Zinn, 1994). Mind wandering was defined as a lack of relation to the current task (Klinger and Cox, 1987; Killingsworth and Gilbert, 2010). Irving (2016) proposed that mind wandering is unguided attention. The main techniques of mindfulness intervention include meditation, body scanning, walking meditation, breathing, and mindfulness yoga (Kabat-Zinn, 2003). Different mindfulness skills are some of the possible specific practices that allow people to understand the core of mindfulness (Kabat-Zinn, 2003). Tang and Posner (2013) proposed that these various mindfulness skills had a common goal, which is being in a state of placidity and awareness of what is occurring within the phenomenological field. More than the conceptual and emotional classifications, mindfulness training requires increasing acceptance of whatever happens and reducing mental judgments.

In recent years, numerous studies have provided empirical evidence for the effectiveness of mindfulness meditation on emotion regulation (e.g., Nyklícek and Kuijpers, 2008; Chambers et al., 2009; Mathew et al., 2010; Geschwind et al., 2011). Researchers have proposed that mindfulness practitioners could be happier and more satisfied (Shapiro et al., 1998, 2008; Ivanovski and Malhi, 2007); be less anxious, less depressive, and have a greater chance of experiencing the feeling of equanimity (Baer et al., 2006); weaken addiction disorder (Bowen et al., 2006); and be healthier (Davidson et al., 2003) than nonmindfulness practitioners. Moreover, some studies have provided evidence that mindfulness training increased cognitive capacity (Brefczynski-Lewis et al., 2007; Jha et al., 2007; Ortner et al., 2007; Tang et al., 2007; McCracken and Yang, 2008) and improved social cognition (Low et al., 2008).

Emotion regulation has been defined as processes through which persons regulate their emotions consciously or unconsciously (Rottenberg and Gross, 2006). Emotional regulation involves many research topics, such as emotional regulation strategies, processes, and outcomes. Individuals use different strategies to modify the intensity or type of emotional experience (Gross, 1998). Successful emotional regulation increases health, improves relationships, and promotes job performance (John and Gross, 2010). Individuals who cannot effectively manage their emotion for a long time may evolve into diagnosable depression or anxiety (Mennin et al., 2007).

However, studies to date on the mechanisms of action underlying mindfulness are inconclusive, and more research is needed. Shapiro et al. (2006) expounded on three mechanisms of mindfulness: intention, attention, and attitude. They posited that intention, attention, and attitude are not separate but are interwoven aspects of mindfulness training and that effective mindfulness training is accompanied by the transformation of these three elements. They proposed that the core potential working mechanism of mindfulness is reperceiving, which promotes the transformation of thinking. Hölzel et al. (2011) suggested that mindfulness training includes a series of different but interrelated compositions which can enhance self-regulation. They proposed four effective compositions: (1) attention regulation, sustaining attention on the chosen object (whenever distracted, returning attention to the object); (2) body awareness, focusing on an object with an internal experience: sensory experiences of breathing, emotions, or other body sensations; (3) emotional regulation, approaching ongoing emotional reactions in a different way and exposing oneself to whatever is present in the field of awareness, letting oneself feel it, and refraining from internal automatic reactivity; and (4) changing the perspective of oneself, separating from a static self-cognition. In addition, they indicated that these four components are similar to the Theravada Buddhist scriptures “Satipatthana Sutta” (mind, body, perception, and law; Hölzel et al., 2011).

“Satipatthana Sutta” is one of the most important references for classic Buddhist mindfulness practice, which requires practitioners not only to have an intention such as compassion for all beings but also stipulating a stringent step-by-step sequence of mindfulness practice. Chiesa (2011) concluded that early stages of mindfulness training are linked with developing the capacity for focusing attention (FA), selecting attention, and executing attention. Later stages of mindfulness training focus on open monitoring (OM) meditation, improving vigilance attention, and keeping attention. Lutz et al. (2008) described two kinds of mindfulness meditation that include FA meditation and OM meditation. FA and OM are also called concentration and mindfulness (Cahn and Polich, 2006). On the one hand, FA meditation requires participants to focus their attention on a chosen object; on the other hand, OM meditation requires participants to monitor the content of their experience in a nonreactive manner from moment to moment. Lutz et al. (2008) conducted a detailed comparison of these two kinds of mindfulness meditation and found that the two kinds of mindfulness meditation had considerable differences in content and features. FA typically requires a relatively narrow field for focusing, such as breathing, while reducing attention to other distracting stimuli. OM, however, needs to make no effort to maintain focus on specific objects. Other researchers have examined the difference between FA and OM (Sears and Kraus, 2009; Perlman et al., 2010). However, few studies have examined the relationship between the training effect and the training order of these two states.

Arch and Craske (2006) found that the focusing breathing group reported lower negative affect and did a more appropriate response to negative stimuli than the unfocused attention and worry group. Perlman et al. (2010) posited that OM has a better effect on individual pain tolerance than FA. However, their study did not provide further exploration of training effects concerning FA and OM. In a similar study, Sears and Kraus (2009) explored the relationship among three groups: FA meditation, OM meditation, and a longer meditation combining both FA and OM meditation. They found that the training effect of the integration group was better than that of the other two groups, indicating that attention and OM in mindfulness training relate to each other and jointly created a better training effect. It should be noted that their study did not differentiate between the longitudinal training sequence effects of FA and OM meditation in the integration group.

Based on the previous findings and Buddhist scripture explanations that FA and OM are interrelated and have a hierarchical relationship (Travis and Shear, 2010), we reasoned that a novice should not directly practice OM before the novice practices FA because going directly to the OM stage would not be effective for beginners. We also thought that FA and OM were equally important. Meditation practitioners might lose the intrinsic meaning of mindfulness if they were not in accordance with the hierarchical relationship of FA and OM.

The present study posited that FA and OM had different working mechanisms and that FA and OM were two kinds of processes for a beginner and emphasized different contents and sequences. Therefore, the purpose of the present study was to investigate the effect of mindfulness training beginning with 4 weeks of FA meditation training, immediately followed by 4 weeks of OM meditation training on emotion regulation. The emotion regulation effects between the 4-week FA meditation and the 4-week OM meditation training were examined. Mind wandering is defined as the transfer of executive control from the attainment of personal goals, often without intention or even awareness of the transfer (Schooler et al., 2004). Mind wandering is the opposite of mindfulness (Smallwood and Schooler, 2006). The level of mind wandering can be used as a measure of mindfulness (Davidson, 2010). We used the Stroop task to measure mind wandering. Meanwhile, Moore and Malinowski (2009) concluded that increased mindfulness are related to improved attentional functions and found that mindfulness meditators performed better at the Stroop task in their study. Moore et al. (2012) further found that mindfulness practices could significantly change neuronal activity related to executive control functions in the Stroop task. We hypothesized that an 8-week mindfulness meditation program would increase mindfulness levels, that FA would significantly decrease depression and anxiety, and that OM would facilitate mindfulness and continue to maintain improvement of the emotional state following FA.

## Materials and Methods

### Participants

The participants consisted of 12 males and 28 females. The mean age of the sample was 22.5 years (range = 19–32 years old). All subjects were healthy and had no smoking or drinking habits. None of the participants had previously practiced mindfulness or other types of meditation. They were randomly assigned into two groups: the training group and the waitlist control group. The participants in the training group took part in mindfulness training for 8 weeks. The participants in the control group were waitlisted for the later training. During the 8-week mindfulness training, four participants in the training group did not finish the training, and thus, there were a total of 16 participant data in the training group and 20 participant data in the control group for data analysis. The experimental procedures were approved by the Institutional Review Board of the State Key Laboratory of Cognitive Neurosciences and Learning of Beijing Normal University.

### Procedure

Participants in the present study were voluntary, and all of them signed an informed consent form for the training and the use of their data. Participants in both the 8-week mindfulness training and the waiting control groups were first introduced to the experiment tasks, which included the questionnaires and the Stroop task. They then completed the experiment tasks at pre- (baseline, the week prior to mindfulness training), mid- (during the 4th and 5th weeks), and post-training (after the 8-week training). The participants in the training group completed 8 weeks of mindfulness training, while the participants in the control group just waited for 8 weeks without mindfulness training. All participants also completed the tasks same as in the baseline during the 4th and 5th weeks, as well as after 8 weeks. After the 8-week training, the participants in the control group participated in the mindfulness training.

### Measures

Questionnaire Materials used in the present study.

#### Freiburg Mindfulness Inventory

Freiburg Mindfulness Inventory (FMI) assesses nonjudgmental present awareness and acceptance of mindfulness. All statements were rated on a four-point scale (1 = seldom; 4 = always). The total score is a summation of all items, ranging from 13 to 52, with higher scores indicating a better mindfulness level (Chen and Zhou, 2014).

#### Positive and Negative Affect Scale

Positive and Negative Affect Scale (PANAS) consists of two subscales in total of 20 items with each subscale consisting of 10 items. One subscale measures positive emotion such as enthusiasm, and the other scale measures negative emotion such as hostility. Each question is rated on a five-point scale that ranges from 1 (never) to 5 (often). The Cronbach’s alpha coefficient of the Chinese version is 0.82 (Huang and Yang, 2003). The participants were asked to fill out the questionnaire according to the experience over the past 1–2 weeks. The total score of the Positive Affect Scale was a summation of the positive items ranging from 10 to 50, with the higher score indicating more positive. The total score of the Negative Affect Scale was a summation of the negative items ranging from 10 to 50, with the higher score indicating more negative.

#### The Beck Anxiety Inventory (Chinese version)

The Beck Anxiety Inventory (BAI) is a measure of anxiety (Beck et al., 1988). The instrument consists of 21 statements rated on a four-point scale (0 = not at all; 3 = severely, I could barely stand it). The Cronbach’s alpha coefficient for the Chinese version of the BAI was 0.95 (Zheng et al., 2002). The summation of all items resulted in a total score, which was converted into a standardized score by the function *Y* = int (1.19×), with the higher score indicating more severe anxiety.

#### The Beck Depression Inventory (Chinese version)

The Beck Depression Inventory (BDI) consists of 21 statements that measure depression (Beck et al., 2009). Each of them was measured on a four-point scale ranging from 0 (not at all) to 3 (severely; I could barely stand it). The Chinese version of the BDI has been shown to be reliable with a Cronbach’s alpha coefficient of 0.89 and a split-half reliability of 0.88 (Zhang et al., 1990). The participants were asked to fill out the questionnaire according to the experience over the past week. The total score was the summation of all items ranging from 0 to 63, with the higher score indicating more severe depression.

#### Rumination-Reflection Questionnaire

The Rumination-Reflection Questionnaire (RRQ) consists of two subscales (Campbell et al., 1996). The Rumination scale measures the recurrent negative self-focus associated with threat or uncertainty. The Reflection scale measures the positive self-focus associated with epistemic interest in the self. Each item is rated on a five-point Likert scale ranging from 1 (strongly disagree) to 5 (strongly agree). The Cronbach’s alpha coefficient of the Chinese version was 0.86 (Yuan et al., 2010). The subscale average score is higher, indicating more rumination or reflection.

##### Cognitive Test: The Stroop Task

The Stroop task requires participants to perform a task of the font color or word meaning inference with the Stroop interference. The task comprised a series of color words including “RED,” “YELLOW,” “BLUE,” and “GREEN” in Chinese characters, which were presented in a matched font color (congruent trial) or a nonmatched font color (incongruent trial). The participants were asked to respond to the specific color of the characters by pressing the specific button as fast and correctly as they could. In the practice block, participants learned to press “D” for red, “F” for yellow, “J” for green, and “K” for blue. Not until the accuracy rate of the participants reached more than 90% in the 24 practice trials could the participants be allowed to enter the experiment trials for using the reaction time (RT) as the only independent variable (Stroop, 1935). There were a total of 154 experiment trials. The half of the trials were congruent color–word pair, while the other half were incongruent color–word pair.

### Mindfulness Intervention

The mindfulness training method in the present study was adopted from the book *Mindfulness: A Practical Guide to Finding Peace in a Frantic World* written by Williams and Penman (2011). The training method was a comprehensive program created by Williams and Penman based on the theory of mindfulness-based stress reduction (MBSR) and mindfulness-based cognitive therapy (MBCT). The program consisted of the 4 weeks of FA meditation practice and then 4 weeks of OM meditation practice. The specific forms of the mindfulness training are in Table 1.

**Table 1:** The specific tasks of mindfulness training program.

Week	Topic	Practices
Week 1	waking up from the autopilot	eat raisin under mindfulness; wake up from routine; habit buster; weekly mindfulness.
Week 2	keeping the body in mind	body scan practice at least 15 min at least twice a day; perform mindfully on another routine activity;habit buster and go for a walk for at least 15 min at least once a week.
Week 3	the mouse in the maze	8 min of movement mindfulness meditation; 8 min of breath and body meditation; 3-min breathing space meditation and practice it twice a day; a habit buster: valuing the television.
Week 4	moving beyond the rumor mill	8-min breath and body meditation; 8-min sound and thought meditation; 3-min breathing space meditation and practice it twice a day.
Week 5	turning toward difficulties	8-min breath and body meditation; 8-min sound and thought mediation; 10-min exploring difficulty meditation; 3-min breathing space meditation.
Week 6	trapped in the past or living in the present	10-min befriending meditation; 3-min breathing space meditation.
Week 7	when did you stop dancing	carry out your own formal meditation practice.
Week 8	your wild and precious life	start the day with mindfulness; use breathing space to punctuate the day; maintain mindfulness practice; befriend your feelings; take a breathing space when you feel tired, frustrated, anxious, anger, or any other powerful motion; mindfulness activities; increase your level of mindfulness exercise; remember the breath.

The mindfulness training program lasted 8 weeks. The training was conducted at 19:00–21:00 PM every Monday for 8 weeks in a psychology laboratory. Participants in the training group were instructed to conduct home mindfulness practice guided by the online training program during the remaining 6 days of the week, 20–30 min per day and to fill the recording sheets. All participants in the training group agreed to complete the home mindfulness practice and record their daily practice on the sheets during the 8 weeks of training.

## Results

### Pre-intervention Analyses

A univariate analysis of variance (ANOVA) was conducted on the mean scores between the mindfulness training and the control group before the mindfulness intervention. There was no significant difference in the mindfulness (scored by FMI) between the training and control groups. In the pre-test, the training group and the control group had no significant differences in positive and negative affect (scored by PANAS), anxiety (scored by BAI), depression (scored by BDI), and rumination (scored by RRQ). However, we found that the mindfulness training and control groups had significant difference in reflection (scored by RRQ; *F*_(1,34)_ = 6.46, *p* = 0.016, *η*^2^ = 0.160), with the training group significantly higher than the control group. In the cognitive test, there were no significant difference in the congruent and incongruent color–word tasks (scored by the Stroop Task). The pre-intervention analyses indicated that the emotional and cognitive levels of participants from two groups were similar. Table 2 shows the descriptive statistics of each score on the three tests for the two groups.

**Table 2:** The descriptive results of the questionnaire and Stroop data at the pre-test, mid-test, and post-test for the training and control groups.

Outcome	Training group (*n =* 16)			Control group (*n =* 20)			*p*-value
	Pre-test *M* (SD)	Mid-test *M* (SD)	Post-test *M* (SD)	Pre-test *M* (SD)	Mid-test *M* (SD)	Post-test *M* (SD)	
FMI	28.75 (4.80)	32.38 (6.18)	34.31 (6.18)	32.00 (4.84)	31.80 (5.95)	31.85 (5.21)	0.009
PANAS Positive	22.81 (4.79)	24.81 (8.08)	25.69 (8.79)	26.20 (8.82)	25.80 (8.82)	24.10 (8.47)	0.031
PANAS Negative	16.94 (7.62)	14.56 (6.50)	15.19 (5.97)	15.95 (4.51)	16.90 (5.59)	16.00 (5.10)	>0.05
BAI	33.88 (12.22)	29.00 (8.77)	30.00 (8.89)	33.20 (8.06)	33.70 (9.95)	31.05 (7.61)	>0.031
BDI	9.06 (8.68)	4.31 (4.81)	4.13 (5.78)	7.90 (5.41)	8.50 (6.31)	6.30 (5.62)	0.005
Rumination	43.06 (7.71)	39.13 (8.14)	38.31 (9.58)	42.00 (5.27)	41.35 (8.63)	42.60 (7.58)	0.008
Reflection	43.31 (6.28)	43.63 (6.22)	44.19 (8.84)	37.90 (6.41)*	37.80 (9.36)	38.55 (8.46)	>0.05
Stroop Congruent	736.56 (172.04)	648.55 (108.01)	660.20 (105.11)	698.05 (101.08)	658.09 (106.29)	671.71 (146.04)	>0.05
Stroop Incongruent	952.51 (254.56)	823.41 (145.80)	849.25 (163.22)	831.75 (136.27)	818.65 (190.19)	808.52 (175.34)	0.042

*Indicates a significant difference between the training and the control groups in the pre-test. What we reported on the *p*-value were *p*-values of the time point × group interaction effect.

### Comparison of Pre-, Mid-, and Post-test Between the Mindfulness Training and Control Groups

The group (training vs. control group) × time (pre- vs. mid-test, post-test) ANOVA analyses were performed for the score of BAI, BDI, FMI, RRQ, PANAS, and Stroop task, respectively.

### Mindfulness

For FMI, the time point × group interaction effect was significant, *F*_(2,68)_ = 5.05, *p* = 0.009, *η*^2^ = 0.129. Simple effect analyses showed a significant difference on the FMI scores among testing sessions (*F*_(2,68)_ = 8.56, *p* < 0.001) in the training group, with the score of the mid-test and post-test being significantly higher than the pre-test (pre-test vs. mid-test, *p* = 0.010; pre-test vs. post-test, *p* = 0.001), whereas there was no significant difference among sessions in the control group.

### Positive and Negative Affect

For PANAS, the results indicated a significant time point × group interaction effect in the positive affect subscale, *F*_(2,68)_ = 3.66, *p* = 0.031, *η*^2^ = 0.097; further simple effect analyses showed that both groups were not significantly different in the three times. On the negative affect subscale, both the main effect and interaction were not significant.

### Anxiety and Depression

For BAI, the results indicated a significant time × group interaction effect, *F*_(2,68)_ = 3.65, *p* = 0.031, *η*^2^ = 0.097. Simple effect analyses of three times data (pre-test, mid-test, and post-test) showed that the BAI score was significantly different in the three tests (*F*_(2,68)_ = 5.79, *p* = 0.005) in the training group, with the score of the mid-test and post-test being significantly lower than that of the pre-test (pre-test vs. mid-test, *p* = 0.002; pre-test vs. post-test, *p* = 0.023). However, no significant difference was found in the control group.

For BDI, there was a significant time × group interaction effect, *F*_(2,68)_ = 5.70, *p* = 0.005, *η*^2^ = 0.144. Simple effect analyses showed a significant difference on the BDI scores among testing sessions (*F*_(2,68)_ = 11.01, *p* < 0.001) in the training group, with the score of the mid-test and post-test being significantly lower than that of the pre-test (pre-test vs. mid-test, *p* = 0.001; pre-test vs. post-test, *p* = 0.001), whereas there was no significant difference among sessions in the control group.

### Rumination–Reflection

On the rumination facet, the time point × group interaction effect was significant (*F*_(2,68)_ = 5.13, *p* = 0.008, *η*^2^ = 0.131), and simple effect analyses showed that the rumination score in each testing time was significantly different (*F*_(2,68)_ = 8.19, *p* = 0.001) in the training group, with the score of the mid-test and post-test being significantly lower than that of the pre-test (pre-test vs. mid-test, *p* = 0.005; pre-test vs. post-test, *p* = 0.001); however, there was no significant difference among sessions in the control group. On the reflection facet, the group main effect was significant (*F*_(1,34)_ = 5.38, *p* = 0.026, *η*^2^ = 0.137); however, the time point × group interaction effect was not significant.

### Stroop Task

For the Stroop task, in the congruent condition, a significant main effect of time was found (*F*_(2,68)_ = 5.35, *p* = 0.007, *η*^2^ = 0.136), showing that the RTs in the mid-test and post-test were significantly lower than that of the pre-test (pre-test vs. mid-test, *p* = 0.003; pre-test vs. post-test, *p* = 0.045). The time point × group interaction effect was not significant. In the incongruent condition, the results indicated a significant time point × group interaction effect, *F*_(2,68)_ = 3.33, *p* = 0.042, *η*^2^ = 0.089. Simple effects showed that the RTs were significantly different (*F*_(2,68)_ = 7.94, *p* = 0.001) among sessions in the training group, with the RTs of the mid-test and post-test being significantly lower than that of the pre-test (pre-test vs. mid-test, *p* = 0.001; pre-test vs. post-test, *p* = 0.013), whereas there was no significant difference among sessions in the control group.

## Discussion

The present study provided empirical evidence that an 8-week mindfulness meditation training program could effectively improve the level of individual mindfulness and the regulation of anxiety, depression, and rumination. Moreover, this study also confirmed that the change of mindfulness level and mood was a dynamic process and FA meditation could partially improve mindfulness level and mood, while OM meditation could further enhance mindfulness level and maintain the effect on mood regulation. It is important to indicate that the mindfulness training methods used in the present study effectively enhanced the mindfulness level. Our findig that the mindfulness training method was effective in improving mindfulness is consistent with findings obtained from previous studies (Nyklícek and Kuijpers, 2008; Farb et al., 2010; Robins et al., 2012).

The present study found that 8-week mindfulness meditation training could effectively regulate mood and reduce anxiety and depression. This finding has been observed in several previous mindfulness studies (Baer et al., 2006; Sears and Kraus, 2009). For example, Sears and Kraus (2009) found that anxiety scores decreased in the FA and OM integrated groups, which is consistent with our study results. However, there was no significant difference in the anxiety scores measured from FA training, which is not consistent with our study. It should be noted that anxiety level in our study was reduced significantly due to the FA training in the first 4 weeks. Further studies are needed to replicate this finding and understand the underlying mechanisms.

Past studies have examined the effectiveness of FA and OM meditation, but no studies have investigated the training effect and order of these two states. For example, Perlman et al. (2010) compared the individual pain tolerance effect of the two stages of FA and OM and found that the effect of OM meditation was better than FA meditation in pain management, without exploring other training effects of FA and OM. In another study, Sears and Kraus (2009) divided participants into three groups: brief meditation focused on attention, brief meditation focused on loving–kindness, and longer meditation combining the attention and loving kindness aspects of mindfulness. Their study found that the third group demonstrated the best effect, which is similar to the finding from the present study. However, Sears and Kraus’s (2009) study did not distinguish whether the sequences of longitudinal research in the third group could cause different results.

The present study also indicated that the RT for the Stroop incongruent tasks was reduced, which reflected the improvement in individuals’ attention levels during mindfulness training. Eight-week mindfulness meditation training also reduced the RT of the individual under the incongruent condition, but not for the congruent condition. This finding was consistent with several previous studies. Moore and Malinowski (2009) reported that compared with the control group, the error rate for the Stroop task had a negative correlation with the increase of the mindfulness level in the meditation group. Their study suggested that meditation training improved the subjects’ ability to complete the Stroop task. However, the subjects in their study were all Buddhist meditators, and the data collection method was a paper test that cannot reach the ideal accuracy. Furthermore, their study did not record RT, which is a reliable indicator for attention level enhanced by mindfulness training. In a similar study, Wenk-Sormaz (2005) discovered that participating in meditation practice could result in a lower interference effect for the Stroop task compared to the control group, indicating that the participants in the meditation group had a stronger anti-interference ability. However, Wenk-Sormaz’s experiment only compared the post-test results of the two groups without comparing the changing results from pre-test to post-test; therefore, the data collected were not sufficiently comprehensive. Similar to our results, Wang et al. (2012) found that mindfulness training improved performance on the Stroop task, mainly in the incongruent condition. However, the major task of the mindfulness training in their experiment was consciously observing one’s own breathing and inner experience of the moment, which involved FA mindfulness and neglected OM mindfulness. We think that the nonsignificant change in RT in the previous studies was most likely attributable to the insufficient training time in which participants were not required to practice every day, except a 10- to 15-min collective exercise per week (Sears and Kraus, 2009). However, the present study required the participants to submit their training records of weekly exercise. In short, compared to previous studies, the present study provided evidence that the combined FA and OM mindfulness training was an effective method to increase participants’ attention and mindfulness by using a methodology that measured the RT and mindfulness at the time of pre-test, mid-test, and post-test.

According to the previous studies and Buddhist scripture, FA and OM meditation are interrelated and have a hierarchical relationship (Peng and Hu, 2011). For participants who have no previous meditation experience, we strongly recommend that they practice FA meditation before OM meditation. In our study, participants with no mindfulness training still obtained a significant positive change in their emotion and cognition in the FA meditation training stage, and OM meditation training further reduced the level of anxiety and depression and improved the participants’ mindfulness level. After 4 weeks of mindfulness training, the experimental results from the present study revealed that compared with the control group, the meditation training group showed enhanced mindfulness levels, reduced RT on the incongruent Stroop tasks, and decreased depression, anxiety, and rumination. After 8 weeks of mindfulness training, the results demonstrated that, compared with the control group, the meditation training group showed further increased mindfulness, reduced RT on the incongruent Stroop tasks, and reduced anxiety, depression, and rumination. This experimental result demonstrated the importance of the FA and OM meditation training sequence. These results also provide empirical evidence that mindfulness training is a gradual process with a specific sequence. Although some monks can naturally achieve the OM state, even without practicing the FA state, for ordinary people, the training effect is cumulative, and it is not easy to directly achieve the OM meditation state just using a simple training program without professional and systematic practices.

In summary, the experimental results in the present study demonstrated that participants showed a gradual improvement in mindfulness during the 8-week training program. For these beginners, FA meditation could significantly reduce the levels of anxiety and depression. Furthermore, OM meditation could further improve mindfulness state and maintain a good mood. However, the pure OM could not be separated in this study. Our study failed to answer the difference in the effect of pure OM and FA, as well as the difference in their working mechanism. This study would be helpful to compare the longitudinal development of FA and OM mindfulness in future studies. Many additional indicators can be used to investigate the training effect, such as event-related potential (ERP), functional magnetic resonance imaging (fMRI), and other physiological indicators. Furthermore, whether FA and OM meditation have different benefits on different abilities is worthwhile to explore, and future studies should also compare the different stages of mindfulness training with relevant information contained in Buddhist classical scriptures. This kind of research based on traditional Buddhist culture may be beneficial in the development of mindfulness training methods.

## Data Availability

The raw data supporting the conclusions of this manuscript will be made available by the authors, without undue reservation, to any qualified researcher.

## Ethics Statement

The experimental procedures were approved by the Institutional Review Board of the State Key Laboratory of Cognitive Neurosciences and Learning of Beijing Normal University.

## Author Contributions

QZ and RZ prepared the manuscript. ZW was in charge of training the participants. XW and RZ came up with this idea and research design. XW, LL and JZ collected the data. XW and LL analyzed the data.

## Conflict of Interest Statement

The authors declare that the research was conducted in the absence of any commercial or financial relationships that could be construed as a potential conflict of interest.

## References

[B2] Arch J. J. Craske M. G. (2006). Mechanisms of mindfulness: emotion regulation following a focused breathing induction. Behav. Res. Ther. 44, 1849–1858. 10.1016/j.brat.2005.12.007 16460668

[B3] Baer R. A. Smith G. T. Hopkins J. Krietemeyer J. Toney L. (2006). Using self-report assessment methods to explore facets of mindfulness. Assessment 13, 27–45. 10.1177/1073191105283504 16443717

[B5] Beck A. T. Epstein N. Brown G. Steer R. A. (1988). An inventory for measuring clinical anxiety: psychometric properties. J. Consult. Clin. Psychol. 56, 893–897. 10.1037/0022-006x.56.6.893 3204199

[B4] Beck D. A. Koenig H. G. Beck J. S. (2009). Depression: causes and treatment. Clin. Geriatr. Med. 14, 765–786. 10.1016/S0749-0690(18)30090-9 9799478

[B6] Bowen S. Witkiewitz K. Dillworth T. M. Chawla N. Simpson T. L. Ostafin B. D.. (2006). Mindfulness meditation and substance use in an incarcerated population. Psychol. Addict. Behav. 20, 343–347. 10.1037/0893-164X.20.3.343 16938074

[B7] Brefczynski-Lewis J. A. Lutz A. Schaefer H. S. Levinson D. B. Davidson R. J. (2007). Neural correlates of attentional expertise in long-term meditation practitioners. Proc. Natl. Acad. Sci. U S A 104, 11483–11488. 10.1073/pnas.0606552104 17596341 PMC1903340

[B8] Cahn B. R. Polich J. (2006). Meditation states and traits: EEG, ERP, and neuroimaging studies. Psychol. Bull. 132, 180–211. 10.1037/0033-2909.132.2.180 16536641

[B9] Campbell J. D. Trapnell P. D. Heine S. J. Katz I. M. Lavallee L. F. Lehman D. R. (1996). Self-concept clarity: measurement, personality correlates, and cultural boundaries. J. Pers. Soc. Psychol. 70, 141–156. 10.1037/0022-3514.70.1.141

[B10] Chambers R. Gullone E. Allen N. B. (2009). Mindful emotion regulation: an integrative review. Clin. Psychol. Rev. 29, 560–572. 10.1016/j.cpr.2009.06.005 19632752

[B11] Chen S. Y. Zhou R. L. (2014). Validation of a chinese version of the freiburg mindfulness inventory-short version. Mindfulness 5, 529–535. 10.1007/s12671-013-0208-8

[B12] Chiesa A. (2011). Improving psychotherapy research: the example of mindfulness based interventions. World J. Methodol. 1, 4–11. 10.5662/wjm.v1.i1.4 25237607 PMC4145556

[B13] Davidson R. J. (2010). Empirical explorations of mindfulness: conceptual and methodological conundrums. Emotion 10, 8–11. 10.1037/a0018480 20141297 PMC4485421

[B14] Davidson R. J. Kabat-Zinn J. Schumacher J. Rosenkranz M. Muller D. Santorelli S. F.. (2003). Alterations in brain and immune function produced by mindfulness meditation. Psychosom. Med. 65, 564–570. 10.1097/01.psy.0000077505.67574.e3 12883106

[B15] Farb N. A. S. Anderson A. K. Mayberg H. Bean J. McKeon D. Segal Z. V. (2010). Minding one’s emotions: mindfulness training alters the neural expression of sadness. Emotion 10, 25–33. 10.1037/a0017151 20141299 PMC5017873

[B16] Geschwind N. Peeters F. Drukker M. van Os J. Wichers M. (2011). Mindfulness training increases momentary positive emotions and reward experience in adults vulnerable to depression: a randomized controlled trial. J. Consult. Clin. Psychol. 79, 618–628. 10.1037/a0024595 21767001

[B17] Gross J. J. (1998). The emerging field of emotion regulation: an integrative review. Rev. Gen. Psychol. 2, 271–299. 10.1037/1089-2680.2.3.271

[B18] Hölzel B. K. Lazar S. W. Gard T. Schuman-Olivier Z. Vago D. R. Ott U.. (2011). How does mindfulness meditation work? Proposing mechanisms of action from a conceptual and neural perspective. Perspect. Psychol. Sci. 6, 537–559. 10.1177/1745691611419671 26168376

[B19] Huang L. Yang T. (2003). Applicability of the positive and negative affect scale in chinese. Chin. Mental. Health J. 17, 54–56.

[B53] Irving Z. C. (2016). Mind-wandering is unguided attention: accounting for the “purposeful” wanderer. Philos. Studies 173, 547–571. 10.1007/s11098-015-0506-1

[B20] Ivanovski B. Malhi G. S. (2007). The psychological and neurophysiological concomitants of mindfulness forms of meditation. Acta Neuropsychiatr. 19, 76–91. 10.1111/j.1601-5215.2007.00175.x 26952819

[B21] Jha A. P. Krompinger J. Baime M. J. (2007). Mindfulness training modifies subsystems of attention. Cog. Affect. Behav. Neurosci. 7, 109–119. 10.3758/cabn.7.2.109 17672382

[B22] John Y. O. Gross S. J. (2010). Healthy and unhealthy emotion regulation: personality processes, individual differences, and life span development. J. Pers. 72, 1301–1334. 10.1111/j.1467-6494.2004.00298.x 15509284

[B23] Kabat-Zinn J. (1994). Wherever You Go, There You Are: Mindfulness Meditation in Everyday life. New York, NY: Hyperion.

[B24] Kabat-Zinn J. (2003). Mindfulness-based interventions in context: past, present, and future. Clin. Psychol. Sci. Pract. 10, 144–156. 10.1093/clipsy/bpg016

[B25] Killingsworth M. A. Gilbert D. T. (2010). A wandering mind is an unhappy mind. Science 330:932. 10.1126/science.1192439 21071660

[B26] Klinger E. Cox W. M. (1987). Dimensions of thought flow in everyday life. Imagin. Cogn. Pers. 7, 105–128. 10.2190/7k24-g343-mtqw-115v

[B27] Low C. A. Stanton A. L. Bower J. E. (2008). Effects of acceptance-oriented versus evaluative emotional processing on heart rate recovery and habituation. Emotion 8, 419–424. 10.1037/1528-3542.8.3.419 18540758

[B28] Lutz A. Slagter H. A. Dunne J. D. Davidson R. J. (2008). Attention regulation and monitoring in meditation. Trends Cogn. Sci. 12, 163–169. 10.1016/j.tics.2008.01.005 18329323 PMC2693206

[B29] Mathew K. L. Whitford H. S. Kenny M. A. Denson L. A. (2010). The long-term effects of mindfulness-based cognitive therapy as a relapse prevention treatment for major depressive disorder. Behav. Cogn. Psychother. 38, 561–576. 10.1017/s135246581000010x 20374671

[B30] McCracken L. M. Yang S. (2008). A contextual cognitive-behavioral analysis of rehabilitation workers’ health and well-being: influences of acceptance, mindfulness, and values-based action. Rehabil. Psychol. 53, 479–485. 10.1037/a0012854

[B31] Mennin D. S. Holaway R. M. Fresco D. M. Moore M. T. Heimberg R. G. (2007). Delineating components of emotion and its dysregulation in anxiety and mood psychopathology. Behav. Ther. 38, 284–302. 10.1016/j.beth.2006.09.001 17697853

[B1] Moore A. Gruber R. Ferose J. Malinowski P. (2012). Regular, brief mindfulness meditation practice improves electrophysiological markers of attentional control. Front. Hum. Neurosci. 6:18. 10.3389/fnhum.2012.00018 22363278 PMC3277272

[B32] Moore A. Malinowski P. (2009). Meditation, mindfulness and cognitive flexibility. Conscious. Cogn. 18, 176–186. 10.1016/j.concog.2008.12.008 19181542

[B33] Nyklícek I. Kuijpers K. F. (2008). Effects of mindfulness-based stress reduction intervention on psychological well-being and quality of life: is increased mindfulness indeed the mechanism? Ann. Behav. Med. 35, 331–340. 10.1007/s12160-008-9030-2 18535870 PMC2517090

[B34] Ortner C. N. M. Kilner S. J. Zelazo P. D. (2007). Mindfulness meditation and reduced emotional interference on a cognitive task. Motiv. Emot. 31, 271–283. 10.1007/s11031-007-9076-7

[B35] Peng Y. Q. Hu H. Y. (2011). Buddhist meditation—a new exploration of psychology methodological research. Psychol. Explor. 31, 297–302.

[B36] Perlman D. M. Salomons T. V. Davidson R. J. Lutz A. (2010). Differential effects on pain intensity and unpleasantness of two meditation practices. Emotion 10, 65–71. 10.1037/a0018440 20141303 PMC2859822

[B37] Robins C. J. Shian L. K. Ekblab A. G. Brantley J. G. (2012). Effects of mindfulness-based stress reduction on emotional experience and expression: a randomized controlled trial. J. Clin. Psychol. 68, 117–131. 10.1002/jclp.20857 22144347

[B38] Rottenberg J. Gross J. J. (2006). When emotion goes wrong: realizing the promise of affective science. Clin. Psychol. Sci. Pract. 10, 227–232. 10.1093/clipsy.bpg012

[B39] Schooler J. W. Reichle E. D. Halpern D. V. (2004). “Zoning-out during reading: evidence for dissociations between experience and meta-consciousness,” in Thinking and Seeing: Visual Meta Cognition in Adults and Children, ed. Levin D. T. (Cambridge, MA: MIT Press), 204–226.

[B40] Sears S. Kraus S. (2009). I think therefore I om: cognitive distortions and coping style as mediators for the effects of mindfulness meditation on anxiety, positive and negative affect, and hope. J. Clin. Psychol. 65, 561–573. 10.1002/jclp.20543 19241400

[B41] Shapiro S. L. Carlson L. E. Astin J. A. Freedman B. (2006). Mechanisms of mindfulness. J. Clin. Psychol. 62, 373–386. 10.1002/jclp.20237 16385481

[B42] Shapiro S. L. Oman D. Thoresen C. E. Plante T. G. Flinders T. (2008). Cultivating mindfulness: effects on well-being. J. Clin. Psychol. 64, 840–862. 10.1002/jclp.20491 18484600

[B43] Shapiro S. L. Schwartz G. E. Bonner G. (1998). Effects of mindfulness-based stress reduction on medical and premedical students. J. Behav. Med. 21, 581–599. 10.1023/A:1018700829825 9891256

[B44] Smallwood J. Schooler J. W. (2006). The restless mind. Psychol. Bull. 132, 946–958. 10.1037/0033-2909.132.6.946 17073528

[B45] Stroop J. R. (1935). Studies of interference in serial verbal reactions. J. Exp. Psychol. 18, 643–662. 10.1037/h0054651

[B47] Tang Y. Y. Ma Y. Wang J. Fan Y. Feng S. Lu Q.. (2007). Short-term meditation training improves attention and self-regulation. Proc. Natl. Acad. Sci. U S A 104, 17152–17156. 10.1073/pnas.0707678104 17940025 PMC2040428

[B46] Tang Y. Y. Posner M. I. (2013). Tools of the trade: theory and method in mindfulness neuroscience. Soc. Cogn. Affect. Neurosci. 8, 118–120. 10.1093/scan/nss112 23081977 PMC3541497

[B48] Travis F. Shear J. (2010). Focused attention, open monitoring and automatic self-transcending: categories to organize meditations from Vedic, Buddhist and Chinese traditions. Conscious. Cogn. 19, 1110–1118. 10.1016/j.concog.2010.01.007 20167507

[B49] Wang Y. Xin T. Liu X. Zhang Y. Lu H. Zhai Y. (2012). Mindfulness can reduce automatic responding: evidences from Stroop task and prospective memory task. Acta Psychol. Sinica 44, 1180–1188. 10.3724/sp.j.1041.2012.01180

[B50] Wenk-Sormaz H. (2005). Meditation can reduce habitual responding. Adv. Mind Body Med. 21, 33–49. 10.1016/j.jep.2005.01.006 20671347

[B51] Williams M. Penman D. (2011). Mindfulness: A Practical Guide to Finding Peace in a Frantic World. London: Piatkus.

[B52] Yuan L. Peng M. Huang J. H. Zhou R. L. (2010). The Chinese version of rumination-reflection questionnaire in college students: validity and reliability. Chin. J. Clin. Psychol. 18, 701–703.

[B54] Zhang Y. X. Wang Y. Qian M. Y. (1990). Study on the reliability and validity of the beck depression scale. Chin. Mental. Health J. 4, 164–168.

[B55] Zheng J. R. Huang Z. R. Huang J. J. Zhuang X. Q. Wang D. B. Zheng S. Y. (2002). A study of psychometric properties, normative scores and factor structure of beck anxiety inventory Chinese version. Chin. J. Clin. Psychol. 10, 4–6.

